# Insight elements of mathematical problem solving in generally gifted and mathematical experts: ERP amplitudes in PO electrodes

**DOI:** 10.3389/fnint.2025.1523334

**Published:** 2025-04-04

**Authors:** Ilana Waisman, Roza Leikin, Mark Leikin

**Affiliations:** Faculty of Education, University of Haifa, Haifa, Israel

**Keywords:** mathematical insight, general giftedness, mathematical expertise, ERP, PO electrodes, mathematical problem solving

## Abstract

School mathematics mainly embraces algorithmic problem solving, pays less attention to strategic reasoning, and rarely contains insightful problem solving. Based on our previous research, we hypothesize that success in solving insight problems correlates strongly with general giftedness, while mathematical expertise is essential for strategy-based problem solving. Furthermore, we employ a phenomenon of greater ERP amplitudes in PO4/8 electrodes associated with insightful problem solving. In this study, 114 high school students (aged 16–18) with varying degrees of general giftedness and mathematical expertise were asked to solve mathematical problem of three distinct type: (1) function problems, whose solutions are memory-based; (2) area problems that necessitate strategic thinking; and (3) insight problems, that necessitate insight for their resolution. The problem solving process was accompanied by ERP recording. We demonstrate that variations in accuracy of solutions and reaction time for correct responses between tasks are influenced by students’ general giftedness and mathematical expertise. Our ERP analyses partly supported our hypotheses regarding the relationship between PO electrode activation, insight-based problem solving processes, and participants’ levels of giftedness and mathematical expertise.

## Introduction

### Insight problem solving and high mathematical abilities

Problem solving is at the cornerstone of all mathematical activity and is a central tool in mathematical learning and teaching (Polya’s, 1945/1973; National Council of Teachers of Mathematics, 2000). Seminal papers and books in mathematics education have emphasized the significance of mathematical problem solving in shaping educational practices (Schoenfeld, 1992; Silver, 1985; Verschaffel et al., 2020). Researchers have explored diverse facets of this domain, including strategies for problem solving (Silver, 1985), the role of mathematical modeling (Kaiser et al., 2011; Lesh and Lehrer, 2003), creativity in problem solving (Leikin and Sriraman, 2017, 2022), and the development of problem solving expertise (Elgrably and Leikin, 2021). Researchers make a distinction between several types of problem solving approaches (Fleck and Weisberg, 2013; Leikin and Guberman, 2023). For example, Novick and Sherman (2003) distinguish between analytical, memory retrieval, and insight-related problem solving. In a similar vein, according to Ervynck (1991), algorithmic, strategic, and insight-related problem solving processes can be considered as three levels of mathematical creativity.

Insight, often referred to as the “Aha! moment,” is crucial for creative solutions. It represents a sudden comprehension that can lead to novel interpretations and solutions (Eysenck and Keane, 2000; Sternberg and Davidson, 1995). Insight problems have straightforward solutions that remain elusive until the critical features are recognized (Metcalfe and Wiebe, 1987; Weisberg, 2015). Solving insight problems requires overcoming familiar ways of thinking and discovering a new perspective (Eysenck and Keane, 2000) that is not rooted in previous experience. In contrast, non-insight problem solving heavily depends on experience or a repertoire of known methods to provide solutions (Liljedahl et al., 2016; Poincaré, 1952; Pretz et al., 2003; Lesh and Zawojewski, 2007). Scholars suggest that the difference between cognitive processing between insight and non-insight problem solving is that insight solutions are based on parallel processing, while experience-based solutions are based on serial processing (Eysenck and Keane, 2000). However, recent research findings highlight that the distinction between insight and non-insight problem solving is more flexible than previously thought. This suggests that even problems traditionally associated with insight can be tackled using systematic and deliberate strategies (Leikin and Guberman, 2023; Rothmaler et al., 2017). Conversely, several studies have suggested that non-insight problems can also elicit the “Aha” experience. This phenomenon occurs when a solution emerges unexpectedly, providing a sudden clarity in connecting a problem with an appropriate solution strategy (Leikin et al., 2016; Leikin and Guberman, 2023; Webb et al., 2018).

The interplay between insight, creativity, and exceptional mathematical ability operates at multiple levels. Professional mathematicians heavily rely on creative thinking, particularly when developing insight solutions to complex mathematical problems (Hadamard, 1945; Ervynck, 1991). This relationship extends to gifted individuals, who demonstrate a natural predisposition toward insightful problem solving. Leikin (2013) reinforces this connection, showing that generally gifted students more readily generate insight solutions when presented with multiple solution tasks. In turn, mathematical expertise manifests distinctly through advanced problem solving capabilities. Expert mathematicians distinguish themselves through deep knowledge foundations, pattern recognition abilities, and strategic problem solving approaches (Krutetskii, 1976; Schoenfeld and Herrmann, 1982).

At the same time, terms describing exceptional mathematical ability—including mathematical giftedness, high ability, high potential, and excellence—lack consistent differentiation in the literature (Leikin, 2020). This conceptual overlap has led to varied identification methods, ranging from traditional academic metrics (high school performance, SAT-M scores) to broader cognitive measures like IQ testing (Amalric and Dehaene, 2016; Koshy et al., 2009; Benbow and Lubinski, 1996; Taub et al., 2008). We employ our theoretical framework that differentiates between mathematical excellence and general giftedness as distinct cognitive abilities. In Leikin and Leikin's (2009) were introduced a two-factor framework that separately assessed general giftedness (G) and excellence in mathematics (EM). This 2 × 2 design allows to systematically examine how these two factors interact and differ. Subsequent neurocognitive studies (Leikin et al., 2013, 2016; Waisman et al., 2014, 2023; Leikin, 2020) have validated this distinction, demonstrating that while G and EM factors may overlap, they engage different neural mechanisms and cognitive processes.

Even though research is progressing regarding solving problems that require insight and complex problems, there are still not enough studies comparing the performance of high school students with different abilities in various types of complex problem solving. Our study aimed to examine EEG activity among students from different ability levels during memory-based and strategy-based problem solving compared to insight problem solving.

### Neurocognitive studies on problem solving and high mathematical ability

Neurocognitive research demonstrates connections between mathematical competence and brain functioning related to different cognitive tasks. According to the neural efficiency hypothesis, higher cognitive functioning is associated with lower brain activation levels (Haier et al., 1988; Neubauer and Fink, 2009). Therefore, more able individuals display less brain activity than average individuals, particularly for tasks involving simple mental operations (Deary, 2000; Jensen, 2006; Neubauer and Fink, 2009). However, several studies suggest the opposite; that is, those with higher mathematical abilities exhibit higher brain activation in several brain regions when compared with individuals with lower mathematical ability (Amalric and Dehaene, 2016; Grabner and De Smedt, 2011; Waisman et al., 2023). This inconsistency may be explained by the fact that task complexity and task familiarity influence brain activation related to mathematical ability (Neubauer and Fink, 2009; Ren and Libertus, 2023; Waisman et al., 2023).

Recent advancements in neuroscience have provided more in-depth insight into the neural mechanisms that underpin complex problem solving, particularly focusing on the neurocognitive processes involved in insight problem solving. Researchers suggest that the frontoparietal network serves as a core brain region for general (Sohn et al., 2004) and mathematical (e.g., Anderson et al., 2011) problem solving processes. In this context, brain activity patterns when solving insight problems fundamentally differ from those during non-insight problem solving (Jung-Beeman et al., 2004; Kounios et al., 2006). Researchers propose that insight problem solving may engage more extensive brain regions or activate different processes within the same areas, thus involving additional cognitive functions compared to more routine, non-insight problem solving (Dietrich and Kanso, 2010; Knoblich et al., 1999). Electrophysiologic studies have further elucidated the temporal dynamics of insight-related neural activity. These studies have identified specific ERP activity, such as the P300 and the N400, that are associated with the detection and integration of unexpected, meaningful information during insight problem solving (e.g., Sprugnoli et al., 2017). For example, Qiu et al. (2008) found that insight problem solving is associated with increased ERP amplitudes over the superior temporal gyrus.

Moreover, it is believed that each brain hemisphere is specialized in processing different cognitive tasks. The left hemisphere specializes in processing language and is more suitable for handling details, while the right hemisphere specializes in spatial processing and takes a holistic perspective (Ornstein, 1997; Gonzalez et al., 2018). A key observation in these studies is the role of the right hemisphere, particularly during insight problem solving tasks. For instance, increased activity in the right anterior superior temporal gyrus, observable as increased power at the PO8 electrode, has been consistently linked with insight problem solving (Jung-Beeman et al., 2004; Shen et al., 2013; Kounios and Beeman, 2014). This heightened activity is believed to facilitate the rapid integration or generation of solutions, leading to the sudden realization or the “Aha! Moment”—a hallmark of insight. This contrasts with non-insight problem solving, where such rapid and spontaneous integration of ideas is less prevalent, emphasizing the distinct cognitive and neural pathways that support different problem solving strategies.

### The research hypotheses

In Leikin et al. (2016), we elaborated upon the previous findings by Jung-Beeman et al. (2004). Based on the findings of Leikin et al. (2016) we raised the hypotheses related to the effects of G and EM factors on the differences between ERP amplitudes at the PO4/8 electrode site as compared to ERP amplitudes at PO3/7 electrodes associated with solving insight problems (Hypotheses H1) and strategy-based problems (Hypotheses H2).

*H1:* When solving Insight problems, the G factor leads to significantly increased ERP amplitudes at PO4/8 electrodes. This effect will be reflected in (a) significantly higher ERP amplitudes at PO4/8 electrodes as compared to ERP amplitudes at PO3/7 electrodes during late potentials at the problem verification stage exhibited by G students, and (b) significantly higher ERP amplitudes at PO4/8 electrodes in G students than the ERP amplitudes at PO4/8 electrodes evoked in NG participants.

*H2:* When solving strategy-based mathematical tasks, the EM factor is associated with significantly increased ERP amplitudes at the PO4/8 electrodes at the problem introduction stage: (a) EM students would exhibit higher ERP amplitudes at PO4/8 electrodes than at PO3/7 electrodes, and (b) EM students exhibit higher ERP amplitudes at PO4/8 electrodes than NEM participants at the problem introduction stage of strategy-based problems.

Furthermore, based on previous studies on memory-based problem solving (Waisman et al., 2023) we raised Hypothesis H3:

*H3:* When solving memory-based tasks, both EM and G factors will not significantly increase ERP amplitudes at the PO4/8 electrodes as compared to ERP amplitudes at PO3/7 electrodes both at problem introduction stage and the answer verification stage.

## Materials and methods

### Participants

In this paper, we report behavioral and ERP findings associated with the problem solving performance of 114 high school students (11^th^ grade—6–18 years old). Following our validated 2 × 2 research group design that identifies mathematical excellence and general giftedness as separate cognitive factors. The sampling procedure was based on two main factors: Excellence in Mathematics (EM factor) was determined by two criteria: a. School mathematics level and scores and b. SAT-M test performance (Zohar, 1990). The students in the EM group belong to the highest 2% group in mathematical performance. General Giftedness (G factor) included students with IQ scores above 130 (identified in 3rd grade) validated by RPMT (Zohar, 1990) score above 90% (top 2% of population) For more details see, for example, Leikin et al. (2016). This sampling design ensures clear differentiation between groups based on both mathematical excellence and general giftedness, with multiple measures used to validate each factor. After the sampling procedure, 114 students, for whom the data was collected without extensive noises, were subdivided into four experimental groups designed with varying combinations of the EM and G factors:

- G-EM group (N = 29, 22 males): Students who are identified as generally gifted and excelling in mathematics.
- G-NEM group (N = 22, 20 males): Students who are identified as generally gifted but do not excel in mathematics.
- NG-EM group (N = 33, 13 males): Students who are not identified as generally gifted but who excel in mathematics.
- NG-NEM group (N = 30, 13 males): Students who are neither identified as generally gifted nor excelling in mathematics.

All participants met strict inclusion criteria: native Hebrew speaking, right-handedness, and normal/corrected vision, with no history of learning disabilities or neurological disorders. Written informed consent was obtained from both participants and their parents. The study received full ethical approval from the Helsinki Committee (Rambam Medical Center), Israel Ministry of Education, and University of Haifa Ethics Committee.

### Materials and procedure

The study consisted of three tests. The first test featured insight problems that required participants to restructure given information to reach solutions. The second test involved comparing areas of geometric figures, with solutions relying on strategic thinking. The third test asked participants to verify whether function equations corresponded to their respective function graphs, a task that required memory activation. These tests are referred to as the Insight test, Area test, and Function test, respectively. Insight, Area, and Function computerized tests were designed using E-Prime software (Schneider et al., 2002).

**Figure 1:**
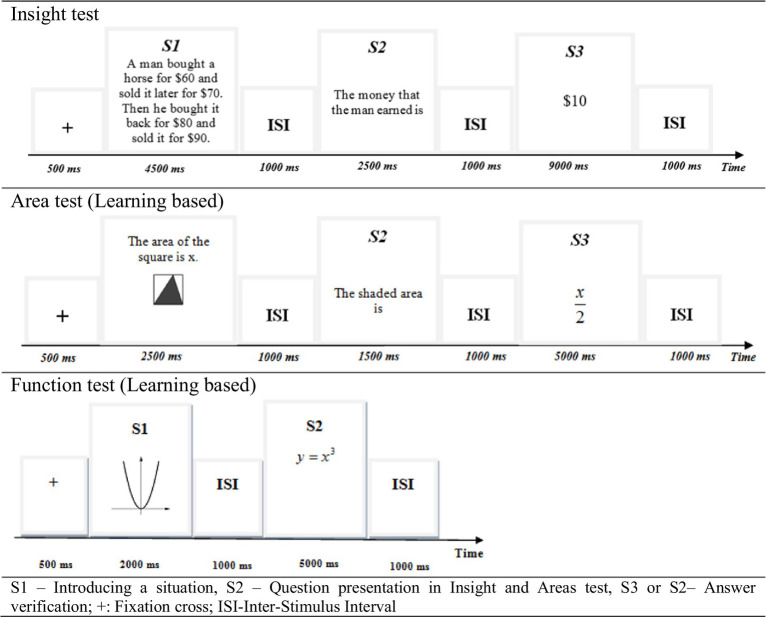
Task examples and sequence of events.

Each test had 60 tasks (trials), with 30 of the 60 tasks depicting a correct answer, while the other 30 tasks depicted an incorrect answer for the task given. All tasks were presented visually at the center of the computer screen, in black characters on a white background (Figure 1). For each task, the students were asked to verify the correctness of the task by pressing the buttons “3” (correct) or “1” (incorrect) according to their decision.

Each task in the Insight and Area tests was presented across three stages: S1 (the presentation of the situation or task condition), S2 (the question presentation), and S3 (the answer verification). These stages appeared consecutively, as presented in Figure 1. In contrast, the Function tasks were presented in two stages: S1 (the presentation of the situation or task condition) and S2 (the answer verification). The division of tasks into these stages is grounded in Polya’s, (1945/1973) theory of problem solving strategies.

The Insight test comprised brief tasks known to elicit an “Aha!” moment (e.g., Wieth and Burns, 2006). During the Area test, participants were provided with a drawing of a geometric figure with a shaded region. They were then asked to verify whether the given formula represented the ratio between the shaded area and the total area of the figure or the ratio between the total area of the figure and its shaded part. For the Function test, participants were shown a graph of a mathematical function followed by an equation and were required to determine whether the graph and the equation represented the same function. This problem solving processing was based on memory retrieval. All three tests demonstrated reliability, with Cronbach’s alpha values exceeding 0.7. The sequence of events and examples of the tasks are presented in Figure 1.

#### EEG recordings

Scalp voltages were continuously recorded using a 64-channel BioSemi Active Two system (BioSemi, Amsterdam, Netherlands) and Active View recording software. Pin-type electrodes were mounted on a customized BioSemi head-cap, arranged according to the 10–20 system. Two flat electrodes were placed on the sides of the eyes to monitor horizontal eye movements. A third flat electrode was placed underneath the left eye to monitor vertical eye movement and blinks. During the session, the electrode offset was kept below 50 μV. The EEG signals were amplified and digitized with a 24-bit AD converter. A sampling rate of 2048 Hz (0.5 ms time resolution) was employed. The Common Mode Sense (CMS) reference and Driven Right Leg (DRL) electrodes were positioned near the midline of the parietal region. Thus, the CMS and DRL electrodes were located to the left and right of the POz channel, respectively.

## Data analysis

### Behavioral data analysis

Analyses were applied to accuracy (Acc) and reaction time for correct responses (RTc). Acc was determined by the participant’s percentage of correct responses to all 60 tasks on the test. RTc was calculated as the mean time spent for verification of an answer (stage S2 for Functions and stage S3 for Areas and Insight) in all correctly solved trials. We analyzed differences in Acc and RTc using analysis of variance (ANOVA) for G (gifted vs. non-gifted) and EM (excelling vs. non-excelling) as between-subject factors. In addition, we examined the differences in Acc and RTc between the tests by using Test (Function, Area, and Insight) as a within-subject factor.

### ERP data analysis

ERP data were preprocessed offline using the Brain Vision Analyzer software (Brain Products). First, ERP data were band pass-filtered between 0.53–30 Hz (zero-phase) and referenced to the common average of all electrodes. Ocular artifacts were corrected using a regression-based approach (Gratton et al., 1983). We divided the continuous ERP data into short non-overlapping epochs of 400 ms duration for the purpose of removing artifacts. We discarded all epochs containing signals exceeding ±80 μV on any channel. The ERP waveforms were time-locked to the onsets of Introducing a situation stage (S1) and Answer verification stage (S2 or S3). The averaged epochs for ERP (including a 200 ms pre-trigger baseline) were 2,200 and 3,200 ms for S1 and S3 at the Insight test, respectively; 1,000 and 3,000 ms for S1 and S3 at the Areas test, respectively; and 1,200 and 2,200 ms for S1 and S2 at Functions test, respectively. The epoch lengths varied across tests to capture the complete duration of cognitive processes specific to each task stage. These differences in epoch lengths aligned with the distinct timing requirements of each test’s stimulus presentation and response windows across the Insight, Area, and Function tests.

For each stage, only epochs for correct responses were averaged. The resulting data were baseline-corrected, and the grand-averaged wave was calculated for each stage. About 40 trials were available for each participant per stage. Based on the visual inspection of the ERP waves, we determined the following time-frames for statistical analysis: Insight test—600–800 ms at S3; Areas-related test—400–500 ms and 600–800 ms at S1; Functions-related test—600-800 ms at S2. The time-frames were chosen based on visual inspection to quantify the difference of the topography in a given time range and our previous studies (Leikin et al., 2016). Afterward, we averaged the channel data into the following regions of interest: Parieto-Occipital left—PO3, PO7, and Parieto-Occipital right—PO4, PO8. In each of the selected time-frames and at each region of interest, we calculated the mean absolute ERP amplitude as an average of the mean absolute ERP amplitude at each electrode within the region.

Between group differences in ERPs (linked the mathematical processing in different groups of participants) were analyzed using repeated measures ANOVA for the mean absolute ERP amplitude, taking G (gifted vs. non-gifted) and EM (excelling vs. non-excelling) as between-subjects factors and Laterality (left vs. right) as within-subjects factor. For all analyses, *p*-values were corrected for deviation from sphericity according to the Greenhouse–Geisser method. Following significant interaction, post-hoc tests using the Bonferroni adjustment were performed.

## Results

### Behavioral measures

Table 1 depicts Acc and RTc, respectively, for the Insight, Area, and Function tests.

**Table 1:** Acc and RTc in the four groups of participants for each test.

			G	NG	Overall
			Mean (SD)		
Acc (%)	Insight	EM	60.2 (9.0)	49.0 (8.8)	54.2 (10.5)
		NEM	54.6 (6.9)	48.8 (7.2)	51.3 (7.6)
		Overall	57.8 (8.5)	48.9 (8.0)	
	Area	EM	83.0 (7.0)	71.0 (8.6)	76.6 (9.9)
		NEM	79.8 (8.0)	73.6 (8.2)	76.2 (8.6)
		Overall	81.6 (7.5)	72.2 (8.5)	
	Function	EM	82.8 (7.4)	76.7 (11.0)	79.6 (9.9)
		NEM	79.1 (7.5)	71.4 (12.8)	74.8 (11.4)
		Overall	81.2 (7.6)	74.2 (12.1)	
RTc (ms)	Insight	EM	1911.1 (478.2)	2500.1 (671.5)	2224.6 (655.2)
		NEM	2401.5 (668.3)	2225.9 (602.6)	2300.2 (630.9)
		Overall	2122.6 (613.0)	2369.5 (649.3)	
	Area	EM	1156.4 (294.0)	1555.7 (503.8)	1369.0 (461.7)
		NEM	1350.5 (371.8)	1284.9 (356.4)	1312.7 (360.9)
		Overall	1240.2 (340.4)	1426.8 (457.2)	
	Function	EM	1544.9 (318.4)	1746.6 (366.2)	1650.8 (356.3)
		NEM	1820.6 (399.6)	1650.2 (427.5)	1724.2 (420.4)
		Overall	1666.8 (379.0)	1700.1 (396.8)	

#### Insight test

**Figure 2:**
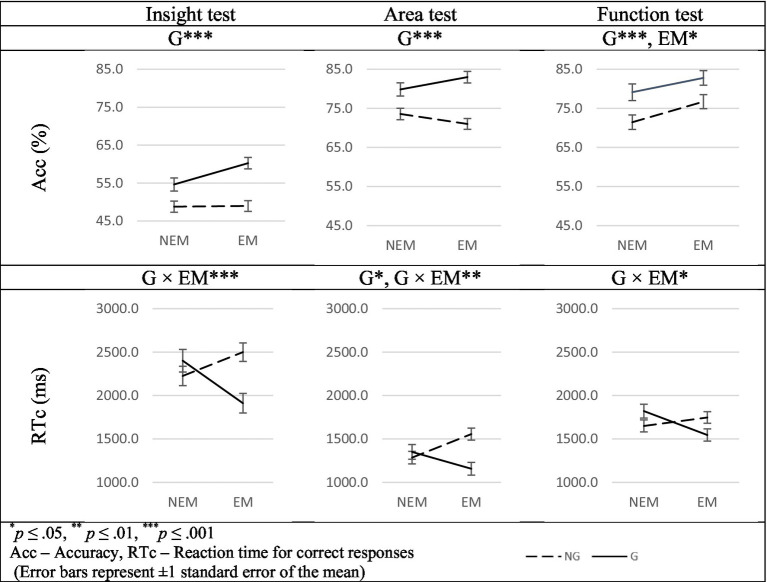
Significant main effects of G and EM factors and G × EM* interactions related to the between-group differences in Acc and RTc.

The main effect of the G factor was found on Acc [*F* (1, 110) = 31.141, *p* < 0.001, $\eta_{p}^{2}$ = 0.221]. G students were more accurate than their NG counterparts (Table 1, Figure 2). In addition, we found a significant interaction of G factor with the EM factor on the RTc [*F* (1, 110) = 10.995, *p* < 0.001, $\eta_{p}^{2}$ = 0.091] (Figure 2). G-EM students were quicker than G-NEM students, while G-NEM students were quicker than their NG-NEM counterparts. Following significant interaction, a post-hoc analysis indicated that the RTc of G-EM students was significantly lower as compared to the RTc of NG-EM students [*F*(1, 110) = 14.458, *p* < 0.001, $\eta_{p}^{2}$ = 0.116] and as compared to G-NEM participants [*F*(1, 110) = 8.122, *p* < 0.01, $\eta_{p}^{2}$ = 0.069].

#### Area test

The main effect of the G factor was found on Acc and RTc. G students were more accurate [*F*(1, 110) = 36.366, *p* < 0.001, $\eta_{p}^{2}$ = 0.248] and quicker as compared to their NG counterparts [*F*(1, 110) = 4.979, *p* < 0.05, $\eta_{p}^{2}$ = 0.043] (Table 1). In addition, we found a significant interaction of the G factor with the EM factor on the RTc [*F*(1, 110) = 9.664, *p* < 0.01, $\eta_{p}^{2}$ = 0.081] (Figure 2). G-EM students were quicker than NG-EM students while G-NEM students were quicker than their NG-NEM counterparts. Following significant interaction, a post-hoc test found that the RTc of G-EM students was significantly lower as compared to the RTc of NG-EM students [*F*(1, 110) = 15.798, *p* < 0.001, $\eta_{p}^{2}$ = 0.126]. In addition, the RTc of NG-EM students was significantly higher as compared to the RTc of NG-NEM participants [*F*(1, 110) = 7.399, *p* < 0.01, $\eta_{p}^{2}$ = 0.063].

#### Function test

The main effect of the EM factor and of the G factor was found on Acc. G students were more accurate as compared to their NG counterparts [*F*(1, 110) = 12.929, *p* < 0.001, $\eta_{p}^{2}$ = 0.105] and EM students were more accurate as compared to their NEM counterparts [*F*(1, 110) = 5.454, *p* < 0.05, $\eta_{p}^{2}$ = 0.047] (Table 1). In addition, we found a significant interaction of G factor with EM factor on RTc [*F*(1, 110) = 6.771, *p* < 0.05, $\eta_{p}^{2}$ = 0.058] (Figure 2). G-EM students were quicker than NG-EM students while G-NEM students were quicker than their NG-NEM counterparts. Following significant interaction, the post-hoc test found that the RTc of G-EM students was significantly lower as compared to the RTc of NG-EM students [*F*(1, 110) = 4.318, *p* < 0.05, $\eta_{p}^{2}$ = 0.036]. In addition, the RTc of G-NEM students was significantly higher as compared to the RTc of G-EM participants [*F*(1, 110) = 6.788, *p* < 0.01, $\eta_{p}^{2}$ = 0.058].

#### Test effect

We found the differences between tests on Acc [*F*(2,214) = 338.746, *p* < 0.001, $\eta_{p}^{2}$ = 0.760] and RTc [*F*(1.836, 196.401) = 6.788, *p* < 0.001, $\eta_{p}^{2}$ = 0.659]. Acc that participants exhibited when solving Insight test was lower as compared to the Acc on Area [*F* (1,110) = 538.878, *p* < 0.001, $\eta_{p}^{2}$ = 0.830] and Function tests [*F*(1,110) = 499.666, *p* < 0.001, $\eta_{p}^{2}$ = 0.818]. At the same time, Acc on the Area test was similar to the Acc on Function tests. RTc on all three tests were significantly different from each other with the highest RTc for the Insight test [Insight vs. Area: *F*(1,110) = 350.425, *p* < 0.001, $\eta_{p}^{2}$ = 0.761, Insight vs. Function: *F*(1,110) = 142.861, *p* < 0.001, $\eta_{p}^{2}$ = 0.563, Function vs. Area tests: *F*(1,110) = 70.311, *p* < 0.001, $\eta_{p}^{2}$ = 0.388].

### Significant effects on ERPs at PO electrodes

**Figure 3:**
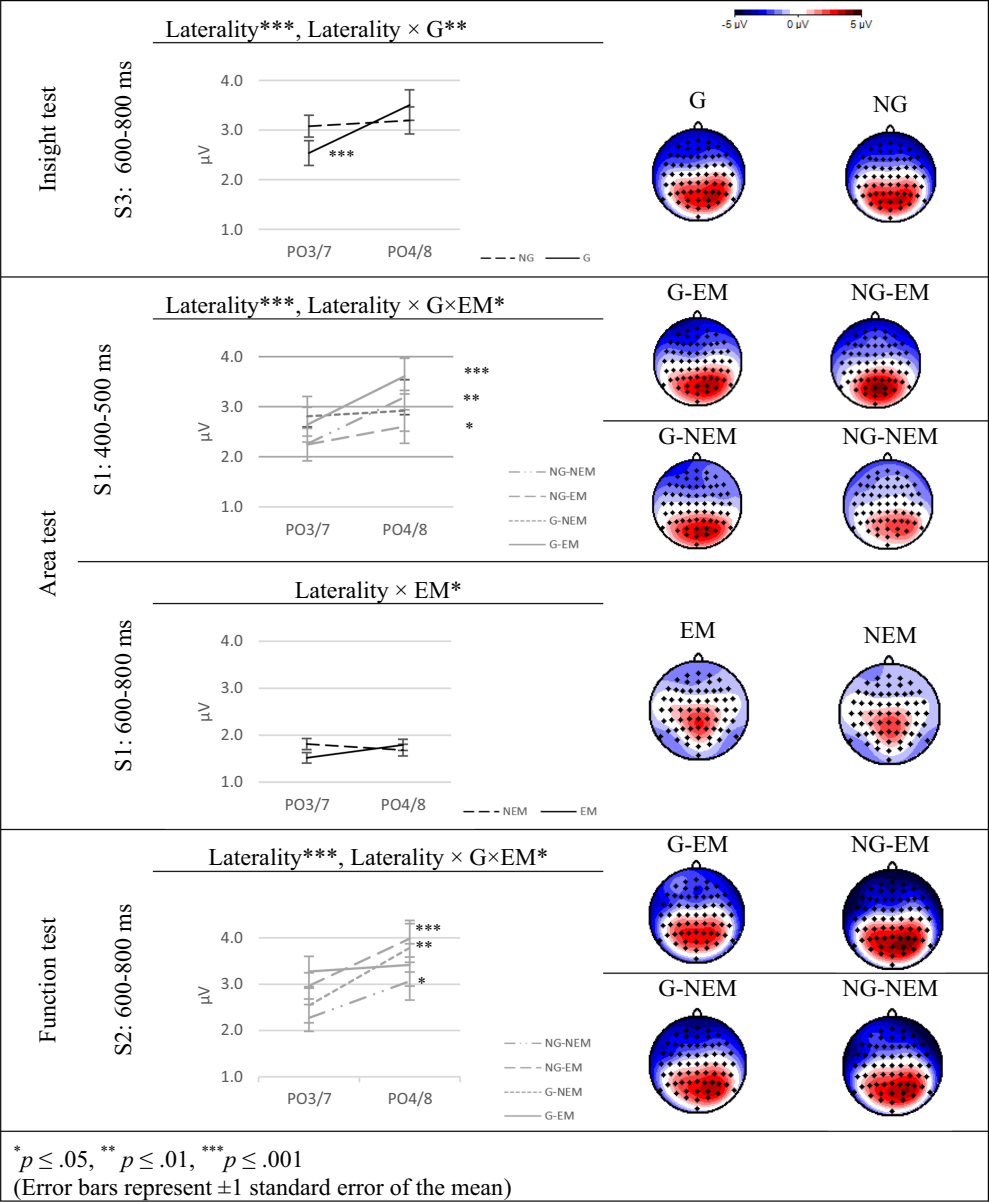
Group effects revealed on ERP mean amplitudes at PO electrodes in the tests of the 3 types.

Figure 3 depicts significant effects found on ERP mean amplitudes at PO electrodes in the tests of the 3 types.

#### Insight test

As described earlier we examined differences in brain activity at PO4/8 vs. PO3/7 electrodes and considered higher ERP amplitudes in PO4/8 linked to insightful problem solving processing. We hypothesized (H1) that G students would exhibit higher ERP amplitudes in PO4/8 electrodes than at PO3/7 electrodes when solving insight problems and that their ERP amplitudes at PO4/8 electrodes would be significantly higher than the ERP amplitudes at PO4/8 electrodes evoked in NG participants when solving insight problems at the S3 stage of solution. This hypothesis was confirmed:

Hypotheses H1 was confirmed: We found that solving insight problems reflected in significantly higher amplitudes at PO4/8 than at PO3/7 electrodes at S3 in the 600–800 ms time-frame [*F*(1,110) = 11.540, *p* < 0.001, $\eta_{p}^{2}$ = 0.095] for all the participants in this study. The effects were mainly caused by the interaction of G factor with Laterality [*F*(1,110) = 7.132, *p* < 0.01, $\eta_{p}^{2}$ = 0.061] (see Figure 3). G students exhibited higher ERP amplitudes at PO4/8 electrodes than did NG participants, whereas at PO3/7 G students exhibited lower ERP amplitudes than did NG participants. That is, the insight component of problem solving characterized G students. The post-hoc test revealed that G students exhibited significantly higher mean amplitudes of PO4/8 electrodes as compared to the mean amplitude at PO3/7 electrodes [*F*(1,50) = 18.153, *p* < 0.001, $\eta_{p}^{2}$ =0.266] while no significant differences in activation of PO4/8 and PO3/7 electrodes was found for NG students.

Following these findings, we used ANOVA to compare difference scores of the mean amplitude on PO4/8 vs. on PO3/7 at the 600–800 ms time frame at S3. As expected, the analysis revealed a significant effect of the G factor [*F*(1,110) = 7.132, *p* < 0.01, $\eta_{p}^{2}$ =0.061]. Specifically, the difference score was significantly larger for the G group compared to the NG group, supporting the findings presented above.

#### Area (strategy-based) test

We hypothesized (H2) that EM students would exhibit higher ERP amplitudes at PO4/8 electrodes than at PO3/7 electrodes and that EM students exhibit higher ERP amplitudes at PO4/8 electrodes than NEM participants at the problem introduction stage of area (strategy-based) problems.

Hypotheses H2 was partially confirmed by the neurocognitive data collected in this study. We found interaction of Laterality with EM factor [*F*(1,110) = 3.946, *p* < 0.05, $\eta_{p}^{2}$ = 0.035] at S1 in the 600–800 ms time-frame (see Figure 3). EM students exhibited higher ERP amplitudes at PO4/8 electrodes than NEM participants, while at PO3/7 EM students exhibited lower ERP amplitudes than NEM participants. This interaction supports H2.

Additionally, for all the participants of this research, solving area problems reflected in Laterality effect at S1 in the 400–500 ms time-frame [*F*(1,110) = 14.836, *p* < 0.001, $\eta_{p}^{2}$ = 0.119]: The mean amplitude at PO4/8 was significantly higher than at PO3/7 electrodes. This finding confirmed our hypothesis related to insightful component linked to solving strategy-based problems. Moreover, the hypothesis was confirmed regarding the ability level of the participants. At S1 in the 400–500 ms time-frame, a significant interaction of Laterality with G and EM factors [*F*(1,110) = 5.421, *p* < 0.05, $\eta_{p}^{2}$ = 0.047] occurred in the 400–500 ms. G-EM participants exhibited higher ERP amplitudes than G-NEM students at PO4/8 electrodes, while at PO3/7 electrodes, G-EM students showed lower ERP amplitudes as compared to their G-NEM counterparts. Additionally, NG-EM participants exhibited lower ERP amplitudes than those of NG-NEM students at PO4/8 electrodes, while the mean amplitude at PO3/7 electrodes was similar for NG-EM and NG-NEM students (see Figure 3). Following this significant interaction effect, further analysis revealed significant interaction of Laterality with EM factor among G students [*F*(1,49) = 4.371, *p* < 0.05, $\eta_{p}^{2}$ = 0.082]: G-EM students exhibited significantly higher ERP amplitudes at PO4/8 as compared to that at PO3/7 electrodes [*F*(1,28) = 13.720, *p* < 0.001, $\eta_{p}^{2}$ = 0.329] while G-NEM students showed similar ERP amplitudes at both PO3/7 and PO4/8 electrodes. These findings demonstrate that at S1 in 400–500 ms, EM factor effects enhanced ERP amplitudes in PO4/8 electrodes among G students only, thus H2 is confirmed for G-EM group of participants.

Following significant effects revealed using ANOVA in the 400–500 ms and 600–800 ms time frames at S1, we conducted a difference score comparison of the mean amplitudes between PO4/8 and PO3/7. As expected, the analysis revealed a significant interaction of the G with EM factors in the 400–500 ms [*F*(1,110) = 5.421, *p* < 0.05, $\eta_{p}^{2}$ =0.047] and a significant effect of EM factor in the 600–800 ms [*F*(1,110) = 3.94, *p* < 0.05, $\eta_{p}^{2}$ =0.035]. These findings demonstrate that for EM students, the differences between amplitudes in PO4/8 and PO3/7 in the 400–500 ms time frame were significantly larger for G-EM participants than for NG-EM participants, while for NEM students the opposite effect was observed: for NG-NEM students the difference was larger than for G-NEM students. This analysis provides additional confirmation of H2.

#### Function (memory-based) test

Hypotheses 3 stated that when solving memory-based tasks, both EM and G factors will not significantly increase ERP amplitudes at the PO4/8 electrodes as compared to ERP amplitudes at PO3/7 electrodes both at problem introduction stage and the answer verification stage. Hypothesis 3 was partially confirmed. We found no significant main effects of G and EM in ERP amplitudes at PO electrodes. Thus. We found significant Laterality effect at S2 at the 600–800 ms time-frame [*F*(1,110) = 24.058, *p* < 0.001, $\eta_{p}^{2}$ = 0.179] as well as a significant triple interaction of Laterality with G and EM factors [*F*(1,110) = 4.153, *p* < 0.05, $\eta_{p}^{2}$ = 0.036] (Figure 3). During the following pair-wise analysis, we found that G-EM students showed similar ERP amplitudes at both PO3/7 and PO4/8 electrodes and this H3 was confirmed only in this group of students. However, other three groups—NG-EM, G-NEM, NG-NEM—exhibited significantly higher ERP amplitudes at PO4/8 electrodes as compared to that at PO3/7 electrodes [*F*(1,32) = 12.848, *p* < 0.001, $\eta_{p}^{2}$ = 0.286; *F*(1,21) = 9.384, *p* < 0.01, $\eta_{p}^{2}$ = 0.309; *F*(1,29) = 5.920, *p* < 0.05, $\eta_{p}^{2}$ = 0.170, respectively].

Following significant ANOVA in the 600–800 ms at S2, we conducted a difference score analysis on the mean amplitude difference between amplitudes in PO4/8 and PO3/7. As expected, this analysis revealed a significant interaction between G with EM factors [*F*(1,110) = 4.153, *p* < 0.05, $\eta_{p}^{2}$ = 0.036] reinforcing findings presented above. We found that the differences for NG-EM participants were larger than for G-EM participants while for G-NEM students the difference was larger than for NG-NEM students. Based on these findings we argue that translation between graphical and symbolic representations of functions was memory-based and easy for G-EM participants. At the same time for students in NG-EM, G-NEM, NG-NEM the task appeared to be more complex, and this complexity is reflected in differences described above.

These findings demonstrate that memory-based processing in the function test occurs in G-EM group only. We assume that higher activation in PO4/8 electrodes in three other groups of participants indicates that memory retrieval was not sufficient for solving function problems for students in these groups and there is an insightful component that helped students to validate that the graph matches formula presented at the introduction stage.

## Discussion

This study aimed to examine the effects of general giftedness and mathematical excellence on the electrical activity at PO electrodes associated with learning-based (area and function) and insight problem solving processing. The 2 × 2 design suggested by Leikin and Leikin (2009) was used to examine the effects of G and EM on accuracy and reaction times at PO electrodes. Compared to our previous studies, which had a purely male sample, the current study includes male and female participants.

### Behavioral effects

Hypothesis H2 was confirmed concerning the accuracy of function tasks but not area tasks. In area tasks, the G factor had the main effect on the accuracy of responses. In addition, no significant main effect of the EM factor on reaction times associated with function and area tasks was observed. These findings align with our earlier studies (Waisman et al., 2014, 2023; Leikin et al., 2016), which likewise reported a main effect of EM factor solely on accuracy for function tasks, without affecting accuracy for area tasks or reaction times in either test.

G students had higher accuracy on all three tests than NG students, consistent with our previous studies (Waisman et al., 2014, 2023; Leikin et al., 2016). Moreover, scholars report that gifted children perform better than their average-achieving peers in insight problem solving. Additionally, we found a significant interaction of both factors associated with learning-and insight tasks. Not surprisingly, giftedness had a significant effect on RTc in EM participants. The G-EM students were much quicker than their NG-EM counterparts on all three tests. The significantly shorter RTc can be explained by efficient information processing (Paz-Baruch et al., 2016; Steiner and Carr, 2003) or by efficient strategies and procedures (Kalyuga, 2008).

The absence of the main effect of the EM factor on the Acc of the area tasks can be attributed to the cognitive characteristics of function and area tasks. It was reported that expertise in algebra involves developing a memory schema for the basic structure of algebraic objects (Geary et al., 2015). Therefore, we argue that students mainly relied on memory during function tasks, whereas area tasks are based on geometrical reasoning that combines visual and logical components (Duval, 1995; Mariotti, 1995). Students’ reasoning about the area of a figure included visual perception of a figure along with thinking about its properties.

### Electrophysiological effects

On the neurocognitive level, we hypothesized that in insight-related test G, students would show enhanced activity at the right PO electrodes at the answer verification stage (S3). In parallel, we hypothesized that in a learning-based test, EM students would show increased activity at the PO4/8 electrodes at the introduction of the problem stage (S1). The time-frame that we used to check our hypothesis was 500–800 ms. This time-frame is connected to the component P600, which may reflect attention-related processing and information integration (Juottonen et al., 1996).

We verified our hypothesis H1 for the Insight test, since significant findings associated with significant interaction with the G factor were found at the answer verification stage (S3). For the area test, significant interactions with the EM factor were found at the problem introduction stage (S1). For the insight test, the mean absolute ERP amplitude was higher at the right PO electrode (PO4, PO8) for G students than for NG participants. In addition, we verified our hypothesis H2 for the area test since the mean absolute ERP amplitude was higher at the left PO electrodes (PO4-PO8) for EM students than for NEM participants.

Scholars argue that insight and non-insight problem solving show different patterns of neural activity (Oh et al., 2020; Jung-Beeman et al., 2004; Kounios and Beeman, 2014; Shen et al., 2018) because of different cognitive processes (Danek and Wiley, 2020; Salvi et al., 2016; Webb et al., 2019). Our findings strengthen this argument by linking differences in ERP amplitudes at PO3/7 vs. ERP amplitudes at PO4/8 electrodes to ability groups differentiated by G and EM factors.

Jung-Beeman et al. (2004) attributed activation of the right PO8 electrode to increased activity in the right anterior superior temporal gyrus when performing insight solving as compared to non-insight solving. We suggest that mathematical insight is a specific characteristic unique to generally gifted students, since we found increased activation at PO4/8 electrodes associated with solving insight problems at the S3 stage. Note that S3, the stage of verification, is associated with the process of elaboration and evaluation of the solution that has been suddenly achieved in the illumination moment (e.g., Weisberg, 2015).

At the same time, the increased activation of right PO electrodes for area test in EM students could be linked to the ability of experts to predict the problem question based on the problem givens (Schoenfeld, 1992). Accordingly, this may be evidence that for experts, there is an insight-related component involved at the stage of understanding the problem when solving experience-based problems.

In addition, for the function test, we found significant findings associated with the G and EM factors at the answer verification stage (S2) that refute our hypothesis. G-EM students had similar ERP amplitudes at PO4/8 as compared to PO3/7 electrodes. This was not the case for G-NEM, NG-EM and G-NEM students, who had significantly higher ERP amplitudes at PO4/8 electrodes. Following our hypothesis about the manifestation of the sudden realization of the solution in the form of higher ERP activity at PO4/8, we found that G-EM students did not show an insightful component on memory-based tasks. We explain this as resulting from G-EM students’ tendency to have stronger long-term and working-memory resources, which allow them to fluently retrieve the necessary information to support their problem solving efforts in algebraic problem solving with function tasks (Tolar et al., 2009; Geary et al., 2015).

We assume the difference between the findings for area and function tests can be explained by the different nature of these two tasks. Area tasks have visual and logical components, while function processing may rely on long-term memory. Acquiring expertise in algebra involves the formation of a memory schema for the basic structure of equations (Sweller and Cooper, 1985; Geary et al., 2015). In parallel, developing a deep understanding of the area of figures requires non-procedural strategies based on more than just the implementation of area formulas (Kospentaris et al., 2011; Runnalls and Hong, 2020).

In conclusion, our study sheds light on the neurocognitive characteristics associated with differences in mathematical processing on insight and learning-based tasks. Mathematical educators and instructional designers should consider these findings in their debates on ability grouping and insight tasks in mathematics curricula.

## Data Availability

The raw data supporting the conclusions of this article will be made available by the authors, without undue reservation.
