# Supplementary material for: Insight elements of mathematical problem solving in generally gifted and mathematical experts: ERP amplitudes in PO electrodes
Source: Front Integr Neurosci. 2025 Apr 4;19:1523334. doi: 10.3389/fnint.2025.1523334 (PMC12006039; doi:10.3389/fnint.2025.1523334)

Insight elements of mathematical problem solving in generally gifted and mathematical experts: ERP amplitudes in PO electrodes

Ilana Waisman, Roza Leikin, Mark Leikin
Faculty of Education, University of Haifa

Appendix 1:

Waveforms of the ERP amplitudes at PO3/O7 and PO4/8 electrodes for G and EM groups in the 3 tests

Insight Test

	EM	G
	(PO4/8 – PO3/7) PO4/8 PO3/7	
S1	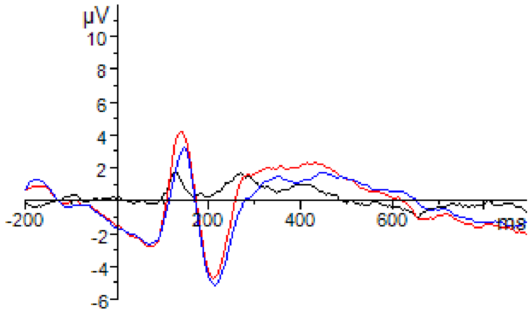	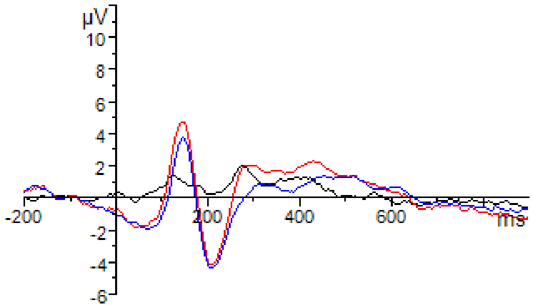
S3	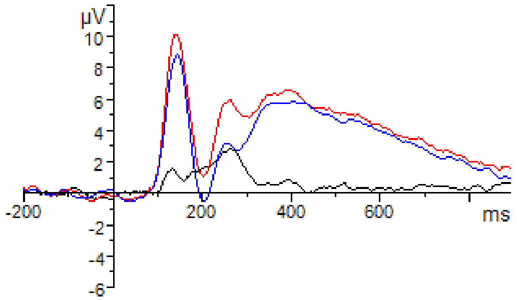	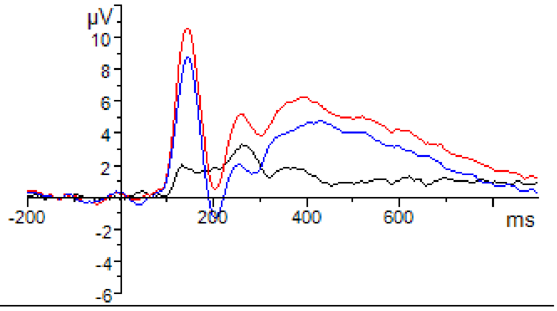

Area - Strategy based - Test

EM	G

(PO4/8 – PO3/7)   PO4/8   PO3/7

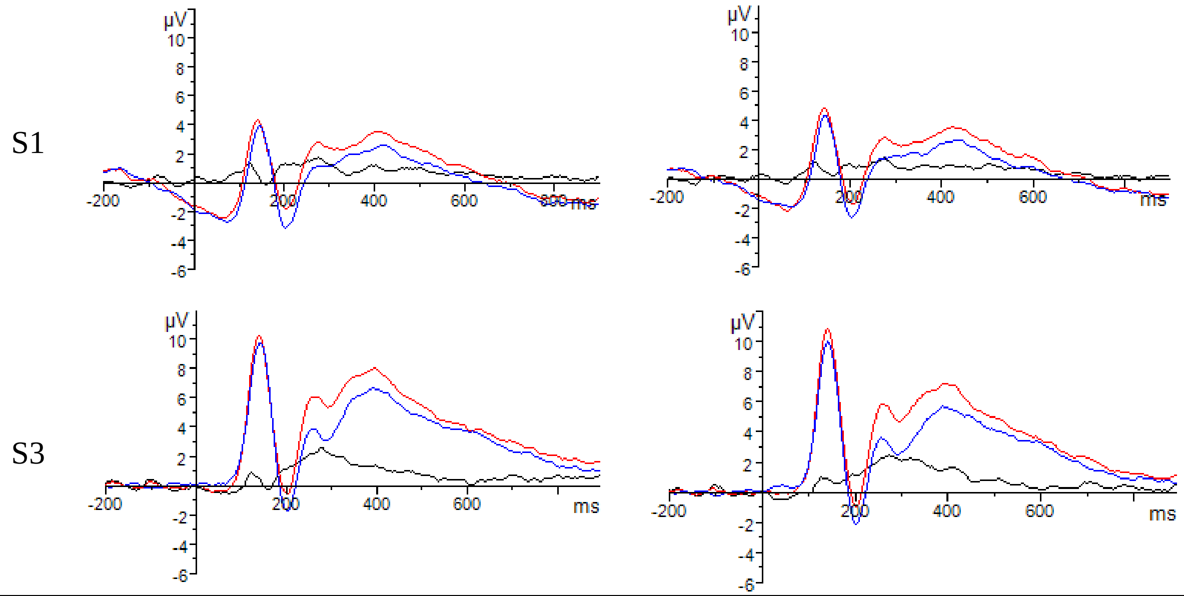

Functions - memory based - test

EM	G

(PO4/8 – PO3/7)   PO4/8   PO3/7

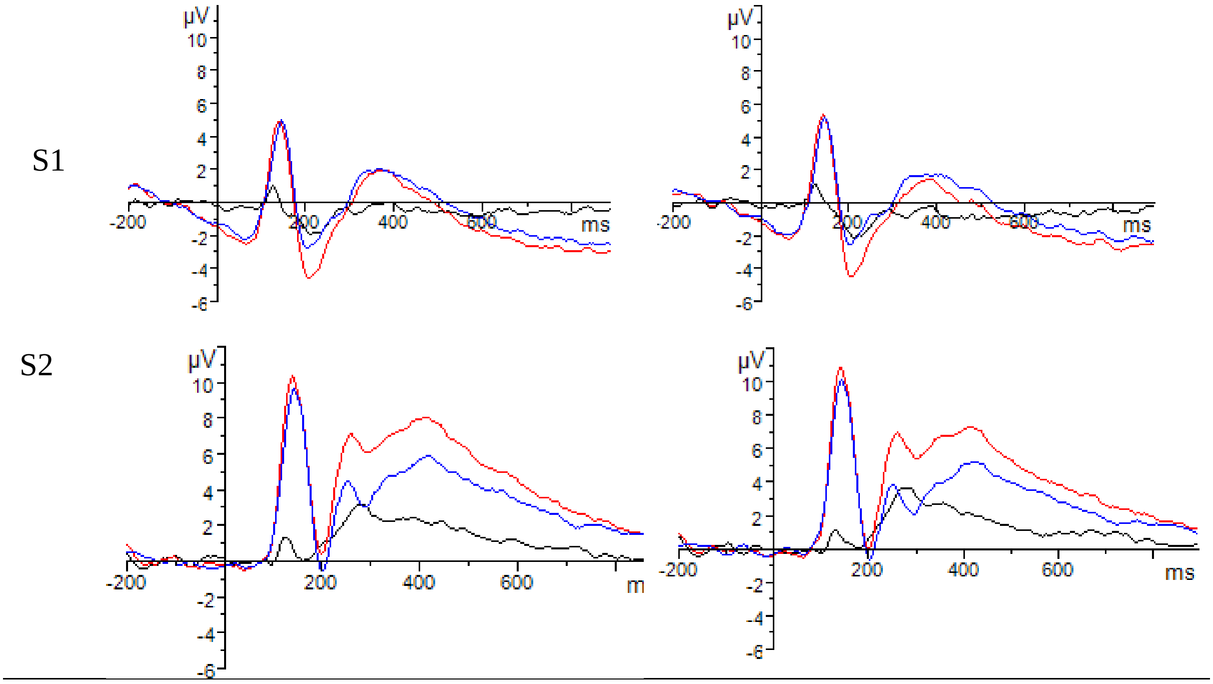

Supplement: Supplementary file 1 [file Supplementary_file_1.pdf]
